# Effect of *Ppd-A1* and *Ppd-B1* Allelic Variants on Grain Number and Thousand Kernel Weight of Durum Wheat and Their Impact on Final Grain Yield

**DOI:** 10.3389/fpls.2018.00888

**Published:** 2018-06-29

**Authors:** Jose M. Arjona, Conxita Royo, Susanne Dreisigacker, Karim Ammar, Dolors Villegas

**Affiliations:** ^1^Sustainable Field Crops Programme, Institute for Food and Agricultural Research and Technology (IRTA), Lleida, Spain; ^2^International Maize and Wheat Improvement Center (CIMMYT), Texcoco, Mexico

**Keywords:** phenology, *Triticum turgidum* L. var. *durum*, photoperiod sensitivity, double ridge, terminal spikelet, yield components

## Abstract

The main yield components in durum wheat are grain number per unit area (GN) and thousand kernel weight (TKW), both of which are affected by environmental conditions. The most critical developmental stage for their determination is flowering time, which partly depends on photoperiod sensitivity genes at *Ppd-1* loci. Fifteen field experiments, involving 23 spring durum wheat genotypes containing all known allelic variants at the PHOTOPERIOD RESPONSE LOCUS (*Ppd-A1* and *Ppd-B1*) were carried out at three sites at latitudes ranging from 41° to 27° N (Spain, Mexico-north, and Mexico-south, the latter in spring planting). Allele *GS100* at *Ppd-A1*, which causes photoperiod insensitivity and results in early-flowering genotypes, tended to increase TKW and yield, albeit not substantially. Allele *Ppd-B1a*, also causing photoperiod insensitivity, did not affect flowering time or grain yield. Genotypes carrying the *Ppd-B1b* allele conferring photoperiod sensitivity had consistently higher GN, which did not translate into higher yield due to under-compensation in TKW. This increased GN was due to a greater number of grains spike^-1^ as a result of a higher number of spikelets spike^-1^. Daylength from double ridge to terminal spikelet stage was strongly and positively associated with the number of spikelets spike^-1^ in Spain. This association was not found in the Mexico sites, thereby indicating that *Ppd-B1b* had an intrinsic effect on spikelets spike^-1^ independently of environmental cues. Our results suggest that, in environments where yield is limited by the incapacity to produce a high GN, selecting for *Ppd-B1b* may be advisable.

## Introduction

Wheat is one of the most widely cultivated crops in the world, with an average annual production, in the last decade, of more than 700 million tons (FAO, 2017). The production forecast for the 2017/18 season is close to 750 million tons, while the consumption is estimated at 720 million tons (FAO, 2017). The expected increase in world population, expected to reach 9.7 billion by 2050, suggests that the global agricultural production has to increase by 25–70% from the current levels (Hunter et al., 2017). This considerable challenge is even greater given the expected climate change scenarios. Therefore, further efforts should be devoted to increasing crop productivity, particularly that of wheat, in regions in which this crop is the most important source of calories and protein for humans. The probability of extreme climate episodes with a large effect on crop productivity, such as drought and heat waves, is increasing (IPCC, 2014). To improve wheat production, a mitigating strategy could be the tailoring of plant development cycles in order to avoid or escape from drought or heat events during the most sensitive phases of yield formation. To this end, among others measures, breeding programs could implement selection for a more efficient and precise phenology, maximizing yield in the prevalent environmental conditions (IPCC, 2014; Hunter et al., 2017).

The main yield components of wheat are grain number per unit area (GN) and thousand kernel weight (TKW), which are therefore important targets in breeding programs. However, the negative correlation between them (Sadras, 2007) limits the breeder’s capacity to increase net yield via the improvement of these two components individually. When a reduction of this negative correlation has been achieved, grain yield (GY) has increased (Griffiths et al., 2015).

Different environmental conditions, during particular developmental phases, affect yield components differently. Low temperature and long pre-flowering periods favor GN (Prasad et al., 2008; Villegas et al., 2016). Temperatures above 31°C around flowering and the first stages of grain filling may affect grain setting, by reducing anther fertility (Draeger and Moore, 2017), thus reducing GN and consequently GY (Ferris et al., 1998; Gibson and Paulsen, 1999; Farooq et al., 2011). Heat stress during grain filling also negatively affects numerous physiological processes, such as membrane stability and metabolism, ultimately causing a reduction in TKW (Farooq et al., 2011). An increase in night temperature from 17 to 23°C has been reported to accelerate grain filling and decrease kernel weight (Prasad et al., 2008). As a result of its negative effect on photosynthesis and starch deposition (Farooq et al., 2011; Rezaei et al., 2015), heat stress reduces nitrogen mobilization efficiency, which is positively correlated with grain weight (Tahir and Nakata, 2005). Therefore, for each particular environment, a balance must be found between a flowering time that is late enough to increase GN but not so late that flowering and grain filling take place under high temperature conditions or terminal drought.

After emergence, wheat development starts with leaf initiation. This vegetative phase ends at the double ridge (DR) stage, giving way to the beginning of the reproductive phase (Slafer and Rawson, 1994). Spikelets start to form from the DR to the terminal spikelet (TS) stages. Floret primordia develop during the stem elongation phase, some becoming actual fertile florets while others degenerate (Kirby and Appleyard, 1986; González et al., 2005). The duration of each phase as well as flowering time, is regulated by vernalization requirement, photoperiod sensitivity and earliness *per se* (Hanocq et al., 2004; Kamran et al., 2014). The PHOTOPERIOD RESPONSE LOCUS (*Ppd-1*) genes belong to the pseudo-response regulators family, which play an important role in controlling circadian cycles, increasing the expression of *CONSTANS* (*CO*) proteins under long days. The CO proteins interact with the *FLOWERING LOCUST T* (*FT*) enhancing their expression and promoting flowering (Valverde et al., 2004). This effect has been found in bread wheat (Beales et al., 2007), and in barley for the *Ppd-H1*, with differences in flowering time between different allelic variants ranging from 7 to 12 days’ difference (Laurie et al., 1994; Turner et al., 2005). In winter barley, a second photoperiod sensitivity gene (*Ppd-H2*) has been characterized and mapped to chromosome 1 (*HvFT3*). The allele conferring insensitivity upregulates vernalization genes and triggers early flowering under short daylength, in some cases even when the vernalization requirements are not fulfilled (Casao et al., 2011).

In spring durum wheat (*Triticum turgidum* L. var. *durum*), two important genes, *Ppd-A1* and *Ppd-B1* (Laurie, 1997; Maccaferri et al., 2008; Wilhelm et al., 2009) on chromosomes 2A and 2B, respectively, have been found to control flowering time through differential response to photoperiod. The *Ppd-A1* gene has three alleles, two of them considered to confer insensitivity (*GS100* and *GS105*), and the wild type allele, which confers sensitivity (*Ppd-A1b*) (Wilhelm et al., 2009). *Ppd-B1* in durum wheat was mapped to the same region as in bread wheat (Maccaferri et al., 2008), and it has only two known alleles, *Ppd-B1a* and *Ppd-B1b*, conferring sensitive and insensitive responses, respectively (Royo et al., 2016). Both genes affect flowering time but to a different extent. The *Ppd-A1* alleles conferring insensitivity cause a greater reduction in the pre-flowering phase duration than *Ppd-B1*, and among the *Ppd-A1* alleles, *GS100* has a stronger effect than *GS105* (Royo et al., 2016). Crop phenology can be adjusted to a certain extent, via the manipulation of photoperiod sensitivity genes, to better fit specific prevailing environmental conditions. Variation in these genes may become a tool for breeders to tailor crop phenology in such a way that the most sensitive developmental phases occur under more favorable conditions.

This study is part of a project designed to analyze the effect of photoperiod sensitivity genes on durum wheat adaptation and productivity. Previous results have recently been published in Royo et al. (2016, 2018) and Villegas et al. (2016). The objective of the present study was to elucidate the effect of photoperiod sensitivity genes *Ppd-A1* and *Ppd-B1* on the formation of the main yield components in durum wheat, namely GN and TKW, and its possible effect on grain yield.

## Materials and Methods

### Plant Material

Twenty-three spring durum wheat genotypes were used in this study (**Supplementary Table S1**). Twenty-one of these lines were derived from crosses between five late flowering genotypes from the breeding program of the University of Hohenheim, Germany [Durabon (*Ppd-A1b, Ppd-B1a*), 2716-25.94.01 (*Ppd-A1b, Ppd-B1a*), Megadur (*Ppd-A1b, Ppd-B1a*), 2805-49.94.02 (*Ppd-A1b, Ppd-B1b*), 2905-13.93.04 (*Ppd-A1b, Ppd-B1a*)] and five early-flowering advanced lines from the CIMMYT-Mexico program [Sooty_9/Rascon_37 (GS-105 *Ppd-A1a, Ppd-B1a*), Cado/Boomer_33 (GS-105 *Ppd-A1a, Ppd-B1b*), Dukem12/2^∗^rascon_21 (GS-100 *Ppd-A1a, Ppd-B1a*), Guanay GS-105 *Ppd-A1a, Ppd-B1b*) and Snitan GS-105 *Ppd-A1a, Ppd-B1b*)]. All crosses were advanced in CIMMYT as bulks without selection up to the F_3_ Generation. Within these, spikes with highly contrasting heading time were selected and advanced as head rows up to the F_8_ generation in Spain. Two well-known commercial cultivars with varying flowering dates were used as controls: Simeto (late-flowering in Mexico and medium to late-flowering in Spain) and Anton (late-flowering in both countries).

### Molecular Characterization

Genotypes were analyzed with a set of molecular markers detailed in Royo et al. (2016). In summary, genotypes were initially characterized for the *Vrn-1* and *Vrn-3* genetic loci (*Vrn-A1, Vrn-B1*, and *Vrn-B3*). Dominant spring alleles were identified in all genotypes on the basis of variation in the promoter and intron-1 region of the *Vrn-A1* locus, which was detected with gene-specific STS markers described by Yan et al. (2004) and Fu et al. (2005).

For *Ppd-A1*, two SNP KASP assays were applied to detect the 1027 bp ‘*GS100*’ type and 1117 bp ‘*GS105*’ type deletion in durum wheat (Wilhelm et al., 2009). For *Ppd-B1*, linked SSR markers gwm148 and gwm257 as described in Hanocq et al. (2004) were used. In addition, gene-specific KASP assays determining truncated copies, transposon-junction, and allele-specific SNPs observed in cv. ‘Sonora64’ (containing three copies of *Ppd-B1*), cv. ‘Chinese Spring’ (carrying four copies of *Ppd-B1*), and cv. ‘Cheyenne’ (carrying one copy of *Ppd-B1*) were tested to determine whether similar allele variation existed in durum wheat (Diaz et al., 2012). However, no copy number variation of *Ppd-B1* alleles was detected. Following Beales et al. (2007), the photoperiod-insensitive allele was designated as *Ppd-1a*. The alternative allele, which was assumed to confer some photoperiod sensitivity, was arbitrarily designed as *Ppd-1b*.

### Experimental Field Setup

**FIGURE 1:**
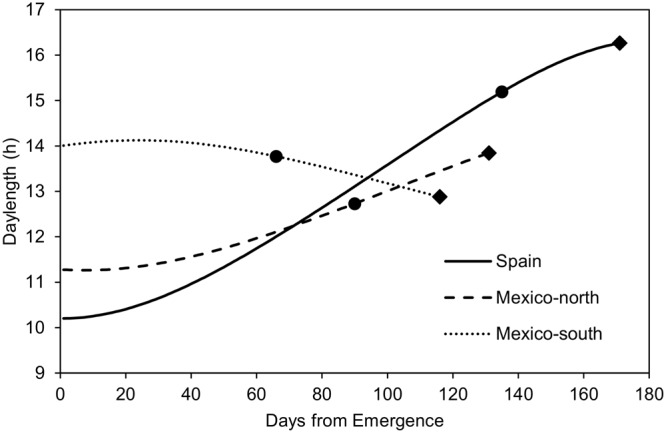
Mean daylength (average of 5 years) during the crop cycles observed at the three testing sites, and mean duration of the emergence-flowering and flowering-maturity phases at each site. Dots: flowering time; Diamonds: maturity time.

The current study involved 15 field experiments that were conducted in 2007, 2008, 2010, 2011, and 2012 at three sites with contrasting latitude: Spain (Gimenells in the north-east), Mexico-north (Ciudad Obregón), and Mexico-south (El Batán Experimental Station in Texcoco, in the Central Mexican Highlands) (**Table 1** and **Supplementary Table S2**). The experiments were arranged in randomized complete block designs with three replications and plots of 12 m^2^. Sowing density was adjusted at each site in order to obtain an approximate plant density of 450 spikes m^-2^. Plots were managed according to the common cultural practices at each site, and were maintained free of weeds, diseases, and pests. Ten experiments were planted in autumn (from November 19 to December 23), while five experiments, corresponding to Mexico-south, were planted in late spring (from May 17 to 28) for a summer crop cycle. Temperatures (absolute maximum and minimum, and mean) and solar radiation (MJ m^-2^ day^-1^) were recorded by meteorological stations placed within or near the experiments. Photoperiod (including twilight) for the emergence-flowering and flowering-maturity periods were calculated according to Forsythe et al. (1995) (**Figure 1**). Full irrigation was provided during the whole cycle in Mexico-north and when necessary to avoid water stress at the other two sites (Spain and Mexico-south).

**Table 1:** Relevant geographic and environmental descriptors for the three testing sites.

Site	Location	Experimental station	Coordinates		Altitude	Long-term	Environmental
		(institution’s acronym)	Latitude	Longitude	(m.a.s.l.)	rainfall (mm/year)	characteristics
Spain	Gimenells (Lleida)	Gimenells (IRTA)	41° 38′N	0° 23′E	200	370	Moderate terminal stress. High to medium productivity
Mexico-north	Ciudad Obregón (Sonora)	CENEB (CIMMYT)	27° 21′N	109° 54′W	40	32	Very high terminal stress. Mandatory full irrigation. Very high productivity
Mexico-south	El Batán (Texcoco)	El Batán (CIMMYT)	19° 31′N	98° 50′W	2249	500	Initial stress eliminated with irrigation. Medium productivity

### Data Recording

In all experiments, the following developmental stages were determined on the central part of each plot according to the Zadoks’ scale (Zadoks et al., 1974): emergence (GS10), flowering (GS65), and physiological maturity (GS87), as indicated by the loss of green color in the spike peduncle. In the experiments conducted in 2010, 2011, and 2012, additional growth stages were determined on each plot: DR and TS (Kirby and Appleyard, 1986), booting (GS45), and heading (GS55). To assess the DR and TS stages, between 3 and 5 plants per plot were sampled 2 to 3 times a week and examined in the laboratory. Leaves were carefully removed, and the main apex of each plant was observed under a binocular magnifier and compared with illustrations in Kirby and Appleyard (1986). A plot was considered to reach the DR or TS stages when 2 out of 3 or 3 out of 5 sampled plants were in the selected stage. A plot was considered to have reached a given developmental stage when at least 50% of the plants exhibited the stage-specific phenotypic characteristics. Thermal time (growing degree-days, GDD) was computed by summing averaged maximum and minimum daily temperatures with 0 and 37°C as base and maximum temperatures, respectively, following Angus et al. (1981).

In all experiments (2007, 2008, 2010, 2011, and 2012), plots were divided into two sections of 6 m^2^, one of which was used for destructive sampling, while the other one was left untouched and was mechanically harvested at commercial maturity. Grain yield (GY, g m^-2^) was obtained, and subsequently adjusted to dry weight basis. TKW (g) was obtained by weighing a randomly drawn sample of 200 kernels from the harvested grain of each plot. The number of grains m^-2^ (GN) was calculated as the ratio of GY to TKW.

Additionally, in experiments performed in 2010, 2011, and 2012, a 1-m-long sample of representative central rows was taken, the spikes were counted and threshed, and their grains were counted. Spikelets spike^-1^ were calculated as the average value of five main spikes randomly chosen on each sample. Grains spike^-1^ was obtained by dividing the number of grains of the sample by the spike number. Grains spikelet^-1^ was calculated as grains spike^-1^ divided by spikelets spike^-1^. Daylength from DR to TS (h) was calculated for each plot in the 2010, 2011, and 2012 experiments by averaging photoperiod between these two developmental stages. Maximum and minimum temperature at flowering (*T*_max_F and *T*_min_F °C, respectively) were determined for each plot as the mean of the maximum or minimum temperatures recorded from 5 days before to 5 days after flowering date.

### Statistical Analysis

Combined ANOVAs were performed across experiments using the GLM procedure of the SAS (2009). statistical package (SAS, RRID:SCR_008567), considering year and genotype as random factors. The sum of squares of the genotype effect was partitioned into differences attributable to allelic variants at *Ppd-A1* and *Ppd-B1* (between allelic classes), and variability between genotypes within allelic classes. The error term used to test *Ppd-1* loci was the sum of squares of genotype within each locus. Means were compared using protected Fisher’s LSD (least significant differences) method at *P* = 0.05, using the sum of squares of genotype within each locus as the error term. A mixed model considering genotype, year and their interactions as random factors was also run using the Kenward-Roger correction, in order to check for the robustness of the significance of the effect of allele variants at *Ppd-A1* and *Ppd-B1* considering the different number of genotypes within each genetic group. Correlation analysis was performed with the pairwise correlation method used by default in JMP (2007) 12 Pro^®^ (JMP, RRID:SCR_014242).

## Results

### Molecular Characterization

**Table 2** shows the allelic composition at the *Ppd-A1* and *Ppd-B1* loci of the 23 genotypes used in this study. A previous study (Royo et al., 2016) showed that all genotypes were spring types, and a more detailed description of the molecular markers used can be found therein.

**Table 2:** Allelic variants at *Ppd-A1* and *Ppd-B1* present in a collection of 23 spring durum wheat genotypes obtained through a divergent selection process for flowering time.

Genes	Alleles^∗^	Photoperiod response	Number of genotypes
*Ppd-B1*			
	*Ppd-B1b*	Sensitive	9
	*Ppd-B1a*	Insensitive	14
*Ppd-A1*			
	*Ppd-A1b*	Sensitive	10
	*GS105*	Insensitive	10
	*GS100*	Insensitive	3

*^∗^Nomenclature described in Wilhelm et al. (2009).*

### Environmental Conditions

**Figure 1** shows the mean photoperiod from emergence to physiological maturity across the 5 years of experiments. In the autumn-sown experiments (Spain and Mexico-north), photoperiod increased during most of the crop cycle. In the late spring planting it increased slightly at the beginning, but decreased during most of the cycle. The mean length of the pre-flowering phase was 1218 GDD (135 days) in Spain, 1440 GDD (90 days in Mexico-north) and 1122 GDD (66 days) in Mexico-south. With regard to the duration from flowering to maturity, Spain had the shortest period with 692 GDD (36 days), followed by Mexico-north with 816 GDD (41 days), and Mexico-south, with 836 GDD (50 days, **Figure 1**).

### Effect of *Ppd-1* Allelic Variants

**FIGURE 2:**
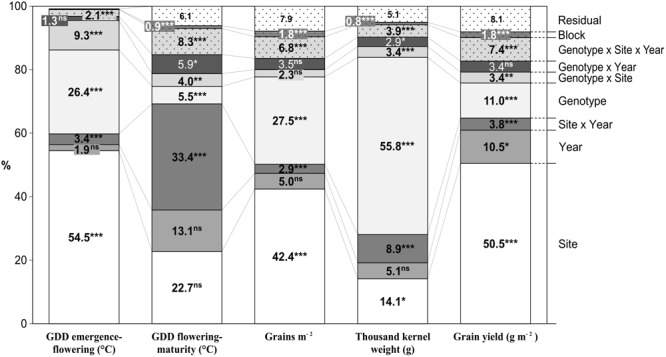
Percentage of the total sum of squares corresponding to the different sources of variation in the ANOVA model obtained from the evaluation of 23 durum wheat genotypes grown in three sites of contrasting latitude during 5 years. ^∗^*P* < 0.05, ^∗∗^*P* < 0.01, ^∗∗∗^*P* < 0.001.

The graphical ANOVA (**Figure 2**) shows that site and genotype were the most important main factors affecting the studied traits, except for thermal time from flowering to maturity, which was affected mostly by the Site × Year interaction. TKW was the least influenced by the site (14.1%), while this source of variation was the most important for all remaining traits except thermal time from flowering to maturity. The site consistently explained a considerably larger proportion of the total variability than the year, the effect of the latter being significant only for yield. Genotypic variation explained a highly variable proportion of the total variability, very little for grain filling period, little in the case of GY, intermediate for GN and pre-flowering thermal time, and very high for TKW. Analyses of variance of the pre-flowering phases were also performed with data of experiments conducted in 2010, 2011, and 2012 (**Supplementary Table S3**).

The genotype effect was further partitioned into differences that could be explained by variation between *Ppd-A1* allelic groups and differences within *Ppd-A1* allelic classes. The same partition was calculated for the *Ppd-B1* locus. The results shown in **Table 3** indicate that differences between genotypes carrying the same allele (within allelic class variation) were generally greater than differences among allelic variants at *Ppd-A1* and *Ppd-B1* loci (between allelic classes variability) (**Table 3**).

**Table 3:** Percentage of the genotype sums of squares from the ANOVA partitioned in differences between allelic variants at *Ppd-A1* and *Ppd-B1* genes and the differences within genotypes carrying a given allele.

Source of variation	d.f.	GDD emergence-flowering	GDD flowering-maturity	Grains m^-2^	Thousand kernel	Grain yield (g m^-2^)
					weight (g)	
**Genotype**	22	26.4^∗∗∗^	5.5^∗^	27.5^∗∗∗^	55.8^∗∗∗^	11^∗∗∗^
		Genotype sum of squares partition by *Ppd-A1*				
Between *Ppd-A1*	*2*	*10.7*^∗∗^	*0.1 ns*	*0.5 ns*	*3.4 ns*	*1.9 ns*
Within *Ppd-A1*	*20*	*15.7*^∗∗∗^	*5.4*^∗∗∗^	*27.0*^∗∗∗^	*52.4*^∗∗∗^	*9.1*^∗∗∗^
		Genotype sum of squares partition by *Ppd-B1*				
Between *Ppd-B1*	*1*	*1.5 ns*	*0.0 ns*	*7.2*^∗^	*10.8*^∗^	*0.0 ns*
Within *Ppd-B1*	*21*	*24.9*^∗∗∗^	*5.5*^∗∗∗^	*20.3*^∗∗∗^	*45.0*^∗∗∗^	*11.0*^∗∗∗^
**Site × Genotype**	44	9.3^∗∗∗^	4.0^∗∗∗^	2.3^∗∗∗^	3.4^∗∗∗^	3.4^∗∗^
		Site × Genotype sum of squares partition by *Ppd-A1*				
Between *Ppd-A1 × Site*	*4*	*2.1*^∗^	*1.5*^∗∗∗^	*0.2 ns*	*0.7 ns*	*0.6 ns*
Within *Ppd-A1 × Site*	*40*	*7.2*^∗∗∗^	*2.5*^∗^	*2.1 ns*	*2.7*^∗∗∗^	*2.8*^∗^
		Site × Genotype sum of squares partition by *Ppd-B1*				
Between *Ppd-B1 × Site*	*2*	*0.2 ns*	*0.3 ns*	*0.0 ns*	*0.2 ns*	*0.6*^∗^
Within *Ppd-B1 × Site*	*42*	*9.1*^∗∗∗^	*3.7*^∗∗∗^	*2.3 ns*	*3.2*^∗∗∗^	*2.8*^∗∗^

*GDD, growing degree-days; ns, non-significant; ^∗^P < 0.05, ^∗∗^P < 0.01, ^∗∗∗^P < 0.001.*

The allelic composition at *Ppd-A1* did not explain any variation in yield, yield components, or grain filling duration. However, it significantly influenced pre-flowering duration. The *Ppd-A1* x Site interaction was significant only for phenology variables.

Allelic differences at *Ppd-B1* explained 7.2 and 10.8% of variations in GN and TKW, respectively, but did not significantly account for the differences observed in GY. On the other hand, the *Ppd-B1* × Site interaction was significant for GY.

Using the mean values of the 23 genotypes at each site across three replicates, and over 5 years, a strong negative correlation was found between GN and TKW at all latitudes, while the correlation between TKW and GY was positive only in Spain. No correlation was found between GN and GY at any of the study sites (**Table 4**).

**Table 4:** Pearson’s correlation coefficients between yield (GY), grain number (GN) and thousand kernel weight (TKW), for experiments involving 23 durum wheat genotypes (*n* = 23) and conducted at 3 sites over 5 years.

	Pearson’s correlation coefficients		
Site	GN-TKW	GN-GY	TKW-GY
Spain	–0.88^∗∗∗^	–0.27 ns	0.68^∗∗∗^
Mexico-north	–0.79^∗∗∗^	0.30 ns	0.32 ns
Mexico-south	–0.72^∗∗∗^	0.40 ns	0.33 ns

*ns, non-significant; ^∗∗∗^P < 0.001.*

At *Ppd-A1*, allele *GS100* was associated with the shortest emergence to flowering period, and *Ppd-A1b* the longest. None of the alleles affected the grain filling period (**Table 5**). The mean maximum temperature to which the crop was exposed 5 days before and after flowering also differed depending on the allele variant at *Ppd-A1*, with the lowest values corresponding to genotypes carrying *GS100* allele. However, in no cases in this study did the temperature reach values that are considered to affect optimal seed set.

**Table 5:** Mean values (and coefficient of variance between brackets) across sites and years for thermal time emergence-flowering (GDD_EF_) and flowering-maturity (GDD_FM_), maximum (*T*_max_F) and minimum temperature around flowering (*T*_min_F), yield components and yield for each *Ppd-A1* and *Ppd-B1* allele.

Gene	Alleles	GDD_EF_ (°C)	GDD_FM_ (°C)	*T*_max_F (°C)	*T*_min_F (°C)	Grains m^-2^	Thousand kernel weight (g)	Grain yield (g m^-2^)
*Ppd-A1*						
	*Ppd-A1b*	1324 (6.6) a	781 (3.4) a	25.5 (1.8) a	9.1 (1.0) b	13903 (2.7) a	41.0 (14.9) a	535 (8.9) a
	*GS105*	1225 (5.5) ab	782 (4.3) a	24.9 (2.2) ab	9.1 (3.6) ab	14493 (17.0) a	40.8 (17.6) a	545 (7.5) a
	*GS100*	1168 (5.3) b	788 (6.4) a	24.6 (1.4) b	9.6 (4.8) a	13991 (16.6) a	45.6 (15.5) a	600 (12.8) a
*Ppd-B1*						
	*Ppd-B1b*	1287 (7.2) a	782 (2.6) a	25.3 (2.3) a	9.3 (4.3) a	15529 (15.6) a	38.0 (16.6) b	549 (10.3) a
	*Ppd-B1a*	1243 (7.6) a	783 (4.8) a	25.0 (2.3) a	9.4 (4.8) a	13299 (11.5) b	43.8 (13.5) a	547 (8.9) a

*T_max_F: mean of the maximum temperatures of 5 days before and after flowering.*

*T_min_F: mean of the minimum temperatures of 5 days before and after flowering.*

*Different letters between alleles at each gene indicate differences according to LSD test at P < 0.05.*

*The number of genotypes carrying each allele is shown in **Table 2**.*

*Ppd-B1* alleles did not affect flowering date, but genotypes carrying the *Ppd-B1b* allele had higher GN with lower TKW than those with *Ppd-B1a*, which resulted in a similar average yield for the two allelic classes (**Table 5**). This *Ppd-B1*-related allelic effect on GN and TKW was consistent at all sites, with the exception of TKW in the summer crop of Mexico-south, for which the difference between the two allelic variants was not statistically significant (**Table 6**). In spite of these consistent differences in GN and TKW between the two allelic classes at *Ppd-B1*, the corresponding difference in yield was not statistically significant at any site.

**Table 6:** Mean values for yield components for each *Ppd-B1* allele.

	Grains m^-2^			Thousand kernel weight (g)			Grain yield (g m^-2^)		
*Ppd-B1*	Spain	Mexico-north	Mexico-south	Spain	Mexico-north	Mexico-south	Spain	Mexico-north	Mexico-south
*Ppd-B1b*	19212a	14135a	13321a	37b	42b	35a	664a	546a	437a
*Ppd-B1a*	17113b	11820b	11001b	43a	48a	40a	693a	538a	412a

*Values are means of experiments conducted over 5 years at each site. Different letters indicate differences according to LSD test at P < 0.05.*

**FIGURE 3:**
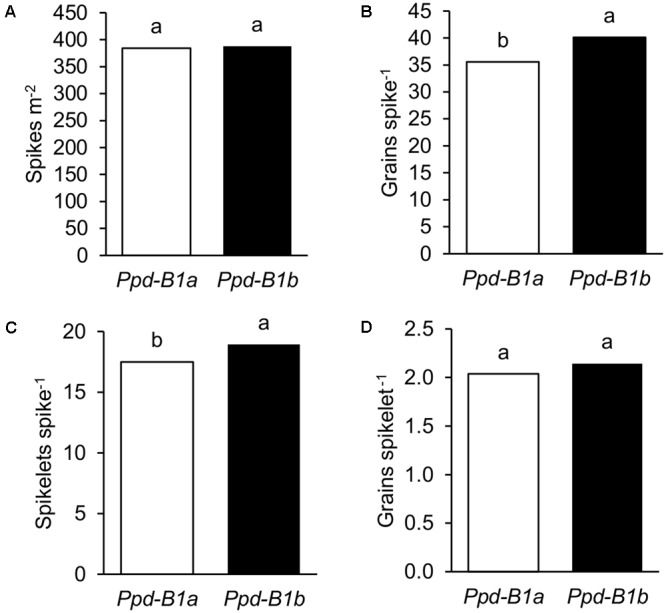
Detailed yield components of 23 durum wheat genotypes grown in three sites of contrasting latitude during 2010, 2011, and 2012: **(A)** spikes m^-2^, **(B)** grains spike^-1^, **(C)** spikelets spike^-1^, and **(D)** grains spikelet^-1^. Each bar represents mean values of genotypes carrying *Ppd-B1a* or *Ppd-B1b*. Different letters indicate differences according to LSD test at *P* < 0.05.

Detailed data on spike characteristics determined in the nine experiments conducted in 2010, 2011, and 2012 were used to elucidate the possible basis underlying the effects of *Ppd-B1* on GN. Results showed that genotypes carrying the *Ppd-B1b* allele had greater GN because they had more grains spike^-1^, since the number of spikes per unit area was similar for the two allelic groups (**Figures 3A,B**). The dissection of grains spike^-1^ into its individual components showed that the number of spikelets spike^-1^ was related to its increase and not the number of grains spikelet^-1^ (**Figures 3C,D**). The effects of *Ppd-B1* on detailed yield components shown in **Figure 3** were also observed at each site independently (**Supplementary Figure S1**).

The length of the pre-flowering phases was analyzed in the nine experiments conducted from 2010 to 2012. Differences between genotypes carrying *Ppd-B1a* and *Ppd-B1b* alleles were not statistically significant for the thermal time of each pre-flowering phase (**Supplementary Tables S4, S5**). Genotypes carrying the *Ppd-B1b* allele tended to have longer phase duration than those with *Ppd-B1a* in all developmental stages except for the duration from heading to flowering (**Supplementary Table S5**), which tended to be slightly longer in genotypes carrying the *Ppd-B1a* allele.

**FIGURE 4:**
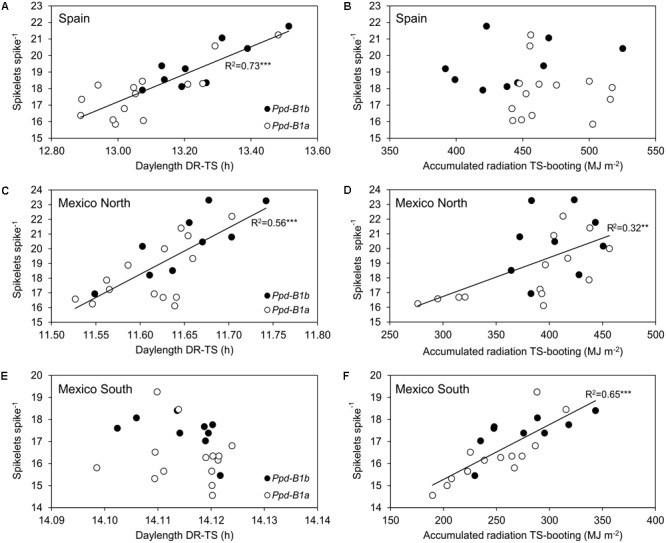
Relationship between mean daylength (h) from double ridge to terminal spikelet **(A,C,E)** and accumulated solar radiation (MJ m^-2^) from terminal spikelet to booting **(B,D,F)** and spikelets spike^-1^ for the 23 durum wheat genotypes carrying the *Ppd-B1a* (∘) and *Ppd-B1b* (•) alleles, for each site across 3 years.

Given that the number of spikelets spike^-1^ is mainly determined in the phase from DR to TS, a linear regression line was fitted for each site to the relationship between the average daylength during this phase and the number of spikelets spike^-1^. The results showed that the average daylength during the DR to TS phase explained 73% of the variation observed in the number of spikelets spike^-1^ in Spain and 56% in Mexico-north (**Figure 4**). However, *Ppd-B1* was significantly associated with differences in daylength during the DR to TS phase only in Spain (*P* < 0.05).

## Discussion

Most studies on the relationship between wheat adaptation and genes that confer photoperiod sensitivity have been conducted in bread wheat (Beales et al., 2007; Diaz et al., 2012; González-Navarro et al., 2015). Only a small number of papers (Maccaferri et al., 2008; Royo et al., 2016) have addressed the effect of *Ppd-1* genes on durum wheat yield and yield components. The current study was designed to unravel the main effects of *Ppd-A1* and *Ppd-B1* alleles on yield and its main components in durum wheat.

Experiments were conducted over 5 years at three contrasting latitudes, a factor that explained a high percentage of total variance in the ANOVA for thermal time from emergence to flowering, GN and GY, and a low, but significant, percentage of the variance for TKW. The influence of the main environmental variables on yield and yield components at the sites included in this study has been previously reported by Villegas et al. (2016).

The genotype effect accounted for a high percentage of the total variance of the model and was significant for all the traits studied. However, the fraction of effects attributable to *Ppd-1* was minor for yield components, but more important for thermal time from emergence to flowering. This result is in agreement with the genetic regulation of phenology and yield components previously stated by several authors (Wang et al., 2011; Kamran et al., 2014; Maphosa et al., 2014). As reported previously by Royo et al. (2016), the effect of *Ppd-A1* on durum wheat phenology was stronger than that of *Ppd-B1*. Alleles conferring photoperiod insensitivity were linked to earlier flowering time, with the *GS100* allele conferring more earliness than *GS105*, in agreement with previous studies (Wilhelm et al., 2009; Royo et al., 2016). The percentage of total variance explained by allelic variants at *Ppd-A1* and *Ppd-B1* (between allelic classes) was small compared with the variation existing between genotypes carrying the same allele (within allelic classes). For phenological traits, this within-classes variation can be attributed to *Eps* genes (Royo et al., 2016). In bread wheat, *Eps* genes have been estimated to be responsible for 5% of the genetic variability for heading time, whenever vernalization and photoperiod genes were also acting (Kamran et al., 2014). In other reports, when vernalization requirements were fulfilled and their effects accounted for, around 50% of genetic variation was attributed to intrinsic earliness (Eagles et al., 2010; Cane et al., 2013). In the case of the genotypes and the environments used in the present study, the percentage of variation attributable to genetic factors unrelated to *Vrn/Ppd* was over 50% for thermal time to flowering. This observation suggests that the influence of putative *Eps* genes on phenology in spring durum wheat would be of the same magnitude as in bread wheat.

The lack of a significant effect of allelic variation at *Ppd-A1* on GN and TKW may be due to the fact that these yield components are regulated by several QTLs (Wang et al., 2011), and the *Ppd-A1* gene act as modifier through the modification of growth cycle length. The substantial genotypic variance for GN and TKW observed in this study (**Figure 2**) further supports this hypothesis. Several authors have reported a strong influence of phenology on GY and have detected additional variation explained by several genomic regions affecting yield and yield components (Maccaferri et al., 2008; Wang et al., 2011; Edae et al., 2014; Kamran et al., 2014; Maphosa et al., 2014).

Early flowering, due to the presence of alleles causing photoperiod insensitivity at *Ppd-A1*, would be expected to affect the yield components as noted by other authors (Slafer et al., 2005; Kamran et al., 2014; Maphosa et al., 2014), with insensitive types increasing yield and yield components at low to medium latitudes, such as those studied here (Kamran et al., 2014; Maphosa et al., 2014, and cites therein). However, in the current study, differences between alleles at *Ppd-A1* were not statistically significant for yield or yield components, even though, numerically, genotypes carrying allele *GS100* yielded 12% more than those carrying the photoperiod sensitive allele *Ppd-A1b*.

However, differences in GN between allelic variants at *Ppd-A1* were small, possibly due to a compensation between the effects caused by alleles conferring photoperiod insensitivity on the potential number of grains and grain setting. It is known that a long pre-flowering period allows the crop to accumulate more biomass at flowering (Royo et al., 2018), produce a high number of grains, and gives it the chance to develop and allocate more resources to reproductive structures (Sinclair and Jamieson, 2006). Accordingly, genotypes carrying the *GS100* allele would have a lower potential number of grains than the sensitive types. On the other hand, early flowering occurring under cooler temperatures is expected to be more favorable for grain filling at low latitudes, where the high temperatures reached during the spring and summer may be limiting for grain setting. The optimal temperatures for flowering are considered to range between 18 and 21°C (Porter and Gawith, 1999), and high temperatures can produce sterility, thereby reducing grain setting (Draeger and Moore, 2017). In the current study, genotypes carrying the *GS100* allele experienced the lowest maximum temperatures at flowering, thus favoring superior grain setting. Therefore, the compensation between the reduction in the potential GN caused by a short pre-flowering period, and the theoretical increase in GN due to a superior grain setting favored by cooler temperatures at flowering could explain the lack of significance of the effect of alleles causing photoperiod insensitivity at *Ppd-A1*on GN.

A shortening of the pre-flowering period is generally associated with a longer flowering-maturity period in some environments (Royo et al., 2016), and TKW could be expected to increase in earlier genotypes due to a longer grain filling period (Joudi et al., 2014). In the current study, differences in the duration of the grain filling period between genotypes with different alleles at *Ppd-A1* were minimal and not significant, with an average of 19 GDD (equivalent to 1 day), and therefore did not have a significant effect on TKW. Nevertheless, genotypes carrying the *GS100* allele produced grains that were, numerically, 11% heavier than those carrying *GS105* and *Ppd-A1b*. As 55.8% of the variation in TKW was explained by the genotype effect, with the sum of the site and year effect and their interactions accounting for 28.1% of the total variation for this trait, our results suggest that genetic factors other than photoperiod sensitivity caused the variations observed in TKW.

The results of the current study indicate that the *Ppd-A1* gene did not have a significant effect on the formation of yield components, but that early-flowering genotypes tended to produce more yield than late ones. The strong effect of *Ppd-A1* on flowering time was not translated into a greater GY, as reported by Maccaferri et al. (2008), who found a few environments across the Mediterranean Basin where early flowering was associated with higher yield. A previous study involving the germplasm used herein demonstrated that the limiting factor for attaining high yield was the capacity of the crop to photosynthesize during the grain filling period (Royo et al., 2018). It is well known that hot and dry conditions after flowering limit the capacity of the crop to support grain filling from transient photosynthesis (Bidinger et al., 1977; Ehdaie et al., 2008; Royo et al., 2018). The current study was conducted under irrigation, thus preventing the drought stress typical of many durum wheat growing environments. Under severe terminal drought stress, early-flowering genotypes would yield significantly more than the late ones, as reported by other authors (Maccaferri et al., 2008; Kamran et al., 2014; Maphosa et al., 2014; Royo et al., 2018, and references therein). In summary, the results of this study suggest that, in the absence of knowledge of other known and well characterized factors, the presence of allele *GS100* could be the most suitable for maximizing yield in environments considered to be close to optimal in terms of water availability.

*Ppd-B1* had a non-significant effect on the flowering time, much smaller than that observed for *Ppd-A1*, in agreement with previous studies (Maccaferri et al., 2008; Royo et al., 2016). Since the effect of *Ppd-B1* on the duration of the emergence-flowering period was not significant, we studied in detail the different phases of this period. The results showed that, when measured in thermal time, none of the phases was significantly different for the two *Ppd-B1* allelic variants, but the *Ppd-B1a* tended to accelerate the initiation of DR stage and the remaining phases with it, as reported in bread wheat (Tanio and Kato, 2007).

However, differences between allelic variants at *Ppd-B1* were significant for both GN and TKW, with photoperiod sensitive genotypes consistently having a higher GN at all sites than the insensitive ones. Therefore, the higher GN achieved by genotypes carrying the *Ppd-B1b* allele would result in higher GY in environments that can satisfactorily sustain the adequate filling of a high number of grains. Genetic gains in GY have been historically been achieved by increasing GN under optimal conditions (Peltonen-Sainio et al., 2007). However, the TKW of genotypes carrying the *Ppd-B1b* allele was proportionally lower, counteracting the effect of higher GN, resulting in a GY equal to that of genotypes carrying the *Ppd-B1a* allele. These results, and the negative correlation between yield components found in the current study and previous ones (Sinclair and Jamieson, 2006; Sadras, 2007; Bustos et al., 2013; Griffiths et al., 2015), exemplify the well documented compensation effect between yield components and the difficulty of breeding for any of them individually and apart with the hope of dramatically increasing. The positive correlation between TKW and GY found in Spain has also been observed previously (García del Moral et al., 2003; Villegas et al., 2016).

A detailed analysis indicated that, when compared with the allele inducing photoperiod insensitivity, the higher GN of genotypes carrying the allele *Ppd-B1b* was not due to a different number of spikes per unit area, but to a greater number of grains spike^-1^ as a result of a greater number of spikelets spike^-1^, as supported by the observation that grains spikelet^-1^ was not affected by *Ppd-B1* allele variation. Lewis et al. (2008) reported a similar effect attributed to *Eps-A^m^1* in *Triticum monococcum* L., where different alleles were found responsible for early development differences linked to the number of spikelets spike^-1^. In their study, however, *EpsA^m^1* was also linked to significant differences in days to heading, as well as differences in days from sowing to DR and from DR to TS, which may constitute a difference between the action of *Ppd-B1* in durum wheat and *Eps-A^m^1* in *T. monococcum*. Edae et al. (2014) found at least two QTLs for number of spikelets spike^-1^ in chormosome 2B. Gao et al. (2015) identified a QTL for floret primordia in the same chromosome, suggesting that additional genes close to *Ppd-B1* may also have a role in determining the final number of spikelets spike^-1^. The direct *Ppd-B1* effect on the number of spikelets spike^-1^ has not been reported in bread wheat. This could be attributed to the important role of the *Ppd-D1* gene (absent in durum wheat) in the determination of spikelets spike^-1^ through control of the expression of *FLOWERING LOCUS T* (*FT*) at early development stages, masking any smaller effect of *Ppd-B1*. Since *Ppd-1* genes are part of the family of pseudo-response regulator (PRR) genes, which affect the circadian clock and control the flowering process (Boden et al., 2015), we hypothesize that, in durum wheat, in the absence of the influence of *Ppd-D1, Ppd-B1* plays a more important role, thus becoming a possible breeding target of choice to increase spikelets spike^-1^ in environments where this change could be advantageous. In bread wheat Maphosa et al. (2014) found that *Ppd-B1b* was associated with a higher GN, and lower TKW, than *Ppd-B1a*, which is consistent with the results obtained in durum wheat in the current study.

Our results suggest that the largest number of spikelets spike^-1^ due to the *Ppd-B1b* allele observed at the three sites had different causes. The initiation of the spikelet primordia occurs around the DR stage, and ends at the TS stage (Porter et al., 1987) and this phase duration was linked to photoperiod in Spain. Moreover, the development of spikelets continues after their initiation in a manner that is influenced by the environment, as observed in both Mexico sites. In Spain, genotypes carrying *Ppd-B1b* reached the DR stage later than those carrying *Ppd-B1a*, and were therefore exposed to a longer photoperiod during part of the DR-TS phase. Accordingly, the high accumulated radiation after this phase did not limit spikelet formation. In contrast, in the spring planting in Mexico-south, daylength during the DR-TS phase was similar for all genotypes regardless of their allelic constitution and radiation from TS to booting therefore must have determined the differences in spikelets spike^-1^. Mexico-north showed an intermediate behavior relative to the other two sites. A longer photoperiod during the DR-TS phase meant more hours of light during spikelet initiation. A possible explanation is that there is a more positive balance between photosynthesis and photorespiration when days are longer during this period, thereby improving the photosynthate source and allowing the plant to increase the sink capacity. However, at both Mexico sites, these environmental effects were unrelated to the significant effect of *Ppd-B1* on final spikelets spike^-1^, since environmental variables shown in **Figure 4** were not significantly different for *Ppd-B1* allelic classes.

Temperature, photoperiod, and their interaction affect the number of spikelets spike^-1^ by determining variations in the duration of the vegetative and early reproductive phases. Slafer and Rawson (1994) reported that the duration from DR to TS decreased with increasing temperature, up to a limit of 19°C. Above this threshold, the duration decreased. They observed the same trend for the number of spikelets spike^-1^. On the other hand, shorter daylength has been associated with a higher number of days from sowing to DR and TS, and more spikelets spike^-1^ (Allison and Daynard, 1976; Miralles et al., 2000). Halse and Weir (1974) considered the interaction between temperature and photoperiod and found that short photoperiods (9 h) were associated with a high number of spikelets spike^-1^, independently of the temperature. They also found that the most favorable temperature during this photoperiod was 15°C, in agreement with Allison and Daynard (1976) and Slafer and Rawson (1994). Our results show that Mexico-north, with a short photoperiod and mild temperatures during the emergence-DR period also had more spikelets spike^-1^ than Mexico-south and Spain. Mexico-south, on the other hand, experienced the longest daylength and showed the smallest number of spikelets spike^-1^. In barley, Ejaz and von Korff (2017) reported an effect of temperature on the regulation of *Ppd-H1*, affecting the shoot apex development. They found that, at the same temperature, the *Ppd-H1* allele conferring sensitivity had an advanced apex development compared with the allele conferring insensitivity, and also reported a qualitative interaction with temperature. A higher number of spikelets spike^-1^ for the *Ppd-B1b* allele was consistently observed at all sites, but durations of pre-flowering phases and meteorological variables in these phases could not explain the effect of *Ppd-B1*. These observations would indicate that, in durum wheat, *Ppd-B1* acts like *Ppd-H1* in barley, as indicated by Ejaz and von Korff (2017).

The results of the present study suggest that *Ppd-B1* intrinsically affects the number of spikelets spike^-1^, independently of environmental conditions. Boden et al. (2015) found relationships between *Ppd-1* genes and spikelet development in wheat, while other authors have located QTLs related to grain number in chromosome 2 close to the *Ppd-B1* position (Gao et al., 2015; Shi et al., 2017). Future studies will be useful to elucidate the exact mechanism underlying the interaction between *Ppd-B1* alleles and the determination of the number of spikelets spike^-1^.

## Author Contributions

CR, KA, and DV designed the experiments and conceived the manuscript. KA and DV purified and increased the germplasm. JA, CR, SD, KA, and DV performed the field evaluations, laboratory determinations and/or data analyses, and read and approved the final manuscript. JA, DV, and CR wrote the manuscript.

## Conflict of Interest Statement

The authors declare that the research was conducted in the absence of any commercial or financial relationships that could be construed as a potential conflict of interest.

## Supplementary Material

FIGURE S1 Detailed yield components of 23 durum wheat genotypes grown in each of three sites of contrasting latitude during 2010, 2011, and 2012: **(A–D)** Spain; **(E–H)** Mexico north; and **(I–L)** Mexico south. Each bar represents mean values of genotypes carrying *Ppd-B1a* or *Ppd-B1b*. Different letters indicate differences according to LSD test at *P* < 0.05.

TABLE S1 Pedigrees and corresponding allelic combinations for *Ppd-A1* and *Ppd-B1* loci present in the genotypes used in the study.

TABLE S2 Environmental variables during the field experiments.

TABLE S3 Percentage of the total sum of squares for pre-flowering phases, corresponding to the different sources of variation in the ANOVA model obtained from the evaluation of 23 durum wheat genotypes grown in three sites of contrasting latitude during three years (2010, 2011, and 2012).

TABLE S4 Percentage of the genotype sums of squares from the ANOVA for pre-flowering phase duration, partitioned in differences between allelic variants at *Ppd-A1 and Ppd-B1* genes and the differences within genotypes carrying a given allele. Data from 3 sites and 3 years.

TABLE S5 Mean values across 3 sites and 3 years for pre-flowering phenological phases for each *Ppd-A1* and *Ppd-B1* allele, expressed as growing degree-days (GDD). The number of genotypes carrying each allele is shown in **Table 2**. Different letters between alleles at each gene indicate differences according to LSD test at *P* < 0.05.

## Abbreviations

DR: double ridge
GN: grain number m^-2^
GY: grain yield (g m^-2^)
QTL: quantitative trait locus
TKW: thousand kernel weight (g)
TS: terminal spikelet
