# Supplementary figures and images for: Effect of Ppd-A1 and Ppd-B1 Allelic Variants on Grain Number and Thousand Kernel Weight of Durum Wheat and Their Impact on Final Grain Yield
Source: Front Plant Sci. 2018 Jun 29;9:888. doi: 10.3389/fpls.2018.00888 (PMC6033988; doi:10.3389/fpls.2018.00888)

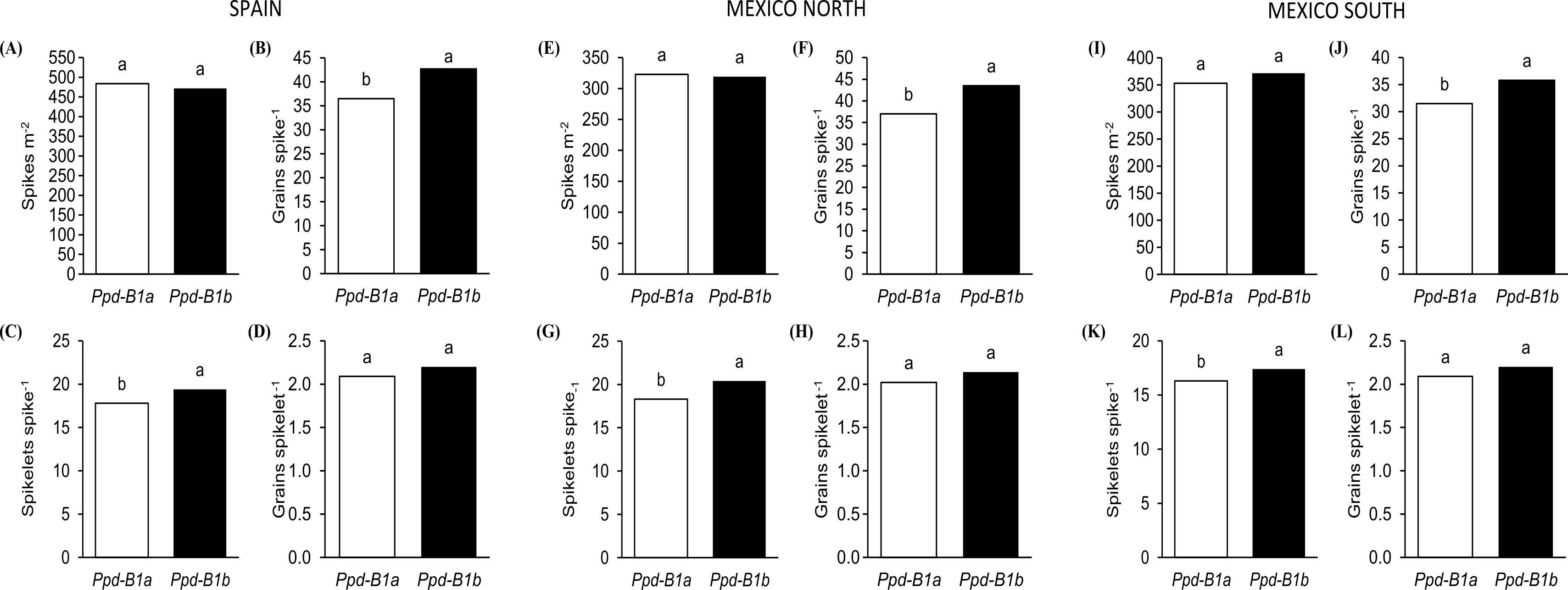

Supplement: FIGURE S1 — Detailed yield components of 23 durum wheat genotypes grown in each of three sites of contrasting latitude during 2010, 2011, and 2012: (A–D) Spain; (E–H) Mexico north; and (I–L) Mexico south. Each bar represents mean values of genotypes carrying Ppd-B1a or Ppd-B1b. Different letters indicate differences according to LSD test at P < 0.05. [file Image_1.TIFF]
