# Supplementary material for: Effect of Ppd-A1 and Ppd-B1 Allelic Variants on Grain Number and Thousand Kernel Weight of Durum Wheat and Their Impact on Final Grain Yield
Source: Front Plant Sci. 2018 Jun 29;9:888. doi: 10.3389/fpls.2018.00888 (PMC6033988; doi:10.3389/fpls.2018.00888)
Supplement: TABLE S1 — Pedigrees and corresponding allelic combinations for Ppd-A1 and Ppd-B1 loci present in the genotypes used in the study. [file Table_1.DOCX]

Supplementary Material

Effect of *Ppd-A1* and *Ppd-B1* Allelic Variants on Grain Number and Weight of Durum Wheat and their Impact on Final Grain Yield

Jose M. Arjona, Conxita Royo, Susanne Dreisigacker, Karim Ammar, Dolors Villegas^*^

***Correspondence:** Dolors Villegas: dolors.villegas@irta.cat

# Supplementary Table

Supplementary table 1. Pedigrees and corresponding allelic combinations for *Ppd-A1* and *Ppd-B1* loci present in the genotypes used in the study.

| Genotype | *Allele at Ppd-A1* | *Allele at Ppd-B1* | Pedigree |
| --- | --- | --- | --- |
| Line 1 | GS-100 *Ppd-A1a* | *Ppd-B1a* | 2905-13.93.04//DUKEM_12/2*RASCON_21 |
| Line 2 | GS-100 *Ppd-A1a* | *Ppd-B1a* | MEGADUR//DUKEM_12/2*RASCON_21 |
| Line 3 | GS-100 *Ppd-A1a* | *Ppd-B1a* | MEGADUR//DUKEM_12/2*RASCON_21 |
| Line 4 | GS-105 *Ppd-A1a* | *Ppd-B1a* | 2716-25.94.01/3/SNITAN |
| Line 5 | GS-105 *Ppd-A1a* | *Ppd-B1a* | 2716-25.94.01/3/SNITAN |
| Line 6 | GS-105 *Ppd-A1a* | *Ppd-B1a* | 2805-49.94.02/GUANAY |
| Line 7 | GS-105 *Ppd-A1a* | *Ppd-B1a* | 2905-13.93.04//CADO/BOOMER_33 |
| Line 8 | GS-105 *Ppd-A1a* | *Ppd-B1a* | DURABON//SOOTY_9/RASCON_37 |
| Line 9 | GS-105 *Ppd-A1a* | *Ppd-B1a* | DURABON//SOOTY_9/RASCON_37 |
| Line 10 | GS-105 *Ppd-A1a* | *Ppd-B1b* | 2805-49.94.02//CADO/BOOMER_33 |
| Line 11 | GS-105 *Ppd-A1a* | *Ppd-B1b* | 2905-13.93.04//CADO/BOOMER_33 |
| Line 12 | GS-105 *Ppd-A1a* | *Ppd-B1b* | 2905-13.93.04//CADO/BOOMER_33 |
| Line 13 | GS-105 *Ppd-A1a* | *Ppd-B1b* | 2905-13.93.04/SNITAN |
| Line 14 | *Ppd-A1b* | *Ppd-B1b* | 2716-25.94.01/GUANAY |
| Line 15 | *Ppd-A1b* | *Ppd-B1b* | 2716-25.94.01/GUANAY |
| Line 16 | *Ppd-A1b* | *Ppd-B1b* | 2905-13.93.04//CADO/BOOMER_33 |
| Line 17 | *Ppd-A1b* | *Ppd-B1b* | 2905-13.93.04//CADO/BOOMER_33 |
| Line 18 | *Ppd-A1b* | *Ppd-B1b* | 2905-13.93.04/SNITAN |
| Line 19 | *Ppd-A1b* | *Ppd-B1a* | 2805-49.94.02//CADO/BOOMER_33 |
| Line 20 | *Ppd-A1b* | *Ppd-B1a* | 2905-13.93.04//CADO/BOOMER_33 |
| Line 21 | *Ppd-A1b* | *Ppd-B1a* | 2905-13.93.04//CADO/BOOMER_33 |
| Anton | *Ppd-A1b* | *Ppd-B1a* | Anton |
| Simeto | *Ppd-A1b* | *Ppd-B1a* | Simeto |
