# Supplementary material for: Effect of Ppd-A1 and Ppd-B1 Allelic Variants on Grain Number and Thousand Kernel Weight of Durum Wheat and Their Impact on Final Grain Yield
Source: Front Plant Sci. 2018 Jun 29;9:888. doi: 10.3389/fpls.2018.00888 (PMC6033988; doi:10.3389/fpls.2018.00888)
Supplement: TABLE S2 — Environmental variables during the field experiments. [file Table_2.DOCX]

Supplementary Material

Effect of *Ppd-A1* and *Ppd-B1* Allelic Variants on Grain Number and Weight of Durum Wheat and their Impact on Final Grain Yield

Jose M. Arjona, Conxita Royo, Susanne Dreisigacker, Karim Ammar, Dolors Villegas^*^

***Correspondence:** Dolors Villegas: dolors.villegas@irta.cat

# Supplementary Table

| **Supplementary Table 2.** Environmental variables during the field experiments | | | | | |  |  |  |
| --- | --- | --- | --- | --- | --- | --- | --- | --- |
|  | **Emergence-flowering** | | |  | **Flowering-maturity** | | | **Total water input during growing season (mm)** |
| **Experiment** | **Minimum temperature (ºC)** | **Maximum temperature (ºC)** | **Mean radiation (MJ m^-2^ day^-1^)** |  | **Minimum temperature (ºC)** | **Maximum temperature (ºC)** | **Mean radiation (MJ m^-2^ day^-1^)** |  |
| Spain 2007 | 3.2 | 13.6 | 11.1 |  | 11.7 | 26.2 | 25.7 | 463 |
| Spain 2008 | 4.4 | 16.4 | 14.2 |  | 11.8 | 23.5 | 21.7 | 640 |
| Spain 2010 | 3.3 | 13.2 | 12.0 |  | 12.0 | 25.5 | 26.0 | 675 |
| Spain 2011 | 3.4 | 15.7 | 13.8 |  | 11.2 | 26.6 | 25.9 | 357 |
| Spain 2012 | 1.9 | 15.2 | 14.0 |  | 13.2 | 28.4 | 26.6 | 299 |
| Mexico North 2007 | 7.0 | 24.1 | 16.2 |  | 9.4 | 29.2 | 24.2 | 384 |
| Mexico North 2008 | 7.1 | 25.4 | 19.7 |  | 11.2 | 32.1 | 27.6 | 507 |
| Mexico North 2010 | 7.7 | 24.5 | 17.0 |  | 9.5 | 28.5 | 25.8 | 444 |
| Mexico North 2011 | 6.4 | 26.0 | 20.0 |  | 11.7 | 31.9 | 26.5 | 420 |
| Mexico North 2012 | 7.0 | 25.2 | 17.4 |  | 8.6 | 28.0 | 25.7 | 404 |
| Mexico South 2007 | 10.3 | 24.9 | 21.4 |  | 10.4 | 23.4 | 18.2 | 670 |
| Mexico South 2008 | 10.3 | 23.2 | 19.3 |  | 10.9 | 23.6 | 18.2 | 482 |
| Mexico South 2010 | 11.0 | 24.5 | 21.0 |  | 10.4 | 22.6 | 18.3 | 637 |
| Mexico South 2011 | 9.0 | 24.7 | 20.7 |  | 8.0 | 24.7 | 21.9 | 525 |
| Mexico South 2012 | 10.7 | 23.9 | 21.2 |  | 10.6 | 23.0 | 19.6 | 489 |
|  |  |  |  |  |  |  |  |  |
