# Supplementary material for: Effect of Ppd-A1 and Ppd-B1 Allelic Variants on Grain Number and Thousand Kernel Weight of Durum Wheat and Their Impact on Final Grain Yield
Source: Front Plant Sci. 2018 Jun 29;9:888. doi: 10.3389/fpls.2018.00888 (PMC6033988; doi:10.3389/fpls.2018.00888)
Supplement: TABLE S3 — Percentage of the total sum of squares for pre-flowering phases, corresponding to the different sources of variation in the ANOVA model obtained from the evaluation of 23 durum wheat genotypes grown in three sites of contrasting latitude during three years (2010, 2011, and 2012). [file Table_3.DOCX]

Supplementary Material

Effect of *Ppd-A1* and *Ppd-B1* Allelic Variants on Grain Number and Weight of Durum Wheat and their Impact on Final Grain Yield

Jose M. Arjona, Conxita Royo, Susanne Dreisigacker, Karim Ammar, Dolors Villegas^*^

***Correspondence:** Dolors Villegas: dolors.villegas@irta.cat

| **Supplementary Table 3.** Percentage of the total sum of squares for pre-flowering phases, corresponding to the different sources of variation in the ANOVA model obtained from the evaluation of 23 durum wheat genotypes grown in three sites of contrasting latitude during three years (2010, 2011 and 2012). | | | | | | | | | | | | | |
| --- | --- | --- | --- | --- | --- | --- | --- | --- | --- | --- | --- | --- | --- |
| **Source** | **d.f.** | **GDD emergence-double ridge (ºC)** | | | **GDD double ridge - terminal spikelet (ºC)** | | **GDD terminal spikelet - booting (ºC)** | | **GDD booting - heading (ºC)** | | | **GDD heading - flowering (ºC)** | |
| Genotype | 22 | 17.9 | *** | 9.6 | | ns | 8.9 | * | | 19.7 | *** | 11.1 | ns |
| Site | 2 | 27.5 | ns | 4.2 | | ns | 26.3 | ns | | 42.0 | * | 13.1 | * |
| Site x Genotype | 44 | 9.2 | *** | 12.6 | | ns | 6.9 | * | | 11.1 | *** | 12.1 | ns |
| Year | 2 | 3.7 | ns | 10.3 | | ns | 8.3 | ns | | 5.9 | ns | 10.2 | * |
| Year x Genotype | 44 | 3.6 | ns | 8.5 | | ns | 5.8 | ns | | 3.7 | ns | 8.3 | ns |
| Site x Year | 4 | 24.4 | *** | 16.8 | | *** | 24.0 | *** | | 9.1 | *** | 2.2 | * |
| Site x Year x Genotype | 88 | 7.1 | *** | 18.8 | | *** | 9.2 | *** | | 4.8 | *** | 18.8 | *** |

GDD: growing degree-days. ns: non-significant; **P*<0.05; ***P*<0.01;****P*<0.001.
