# Supplementary material for: Effect of Ppd-A1 and Ppd-B1 Allelic Variants on Grain Number and Thousand Kernel Weight of Durum Wheat and Their Impact on Final Grain Yield
Source: Front Plant Sci. 2018 Jun 29;9:888. doi: 10.3389/fpls.2018.00888 (PMC6033988; doi:10.3389/fpls.2018.00888)
Supplement: TABLE S4 — Percentage of the genotype sums of squares from the ANOVA for pre-flowering phase duration, partitioned in differences between allelic variants at Ppd-A1 and Ppd-B1 genes and the differences within genotypes carrying a given allele. Data from 3 sites and 3 years. [file Table_4.DOCX]

Supplementary Material

Effect of *Ppd-A1* and *Ppd-B1* Allelic Variants on Grain Number and Weight of Durum Wheat and their Impact on Final Grain Yield

Jose M. Arjona, Conxita Royo, Susanne Dreisigacker, Karim Ammar, Dolors Villegas^*^

***Correspondence:** Dolors Villegas: dolors.villegas@irta.cat

# Suplementary Table

**Supplementary Table 4.** Percentage of the genotype sums of squares from the ANOVA for pre-flowering phase duration, partitioned in differences between allelic variants at *Ppd-A1 and* *Ppd-B1* genes and the differences within genotypes carrying a given allele. Data from 3 sites and 3 years.

| **Source of variation** | **d.f.** | **GDD emergence-double ridge** | | **GDD double ridge-terminal spikelet** | | **GDD terminal spikelet-booting** | | **GDD booting-heading** | | **GDD heading-flowering** | |
| --- | --- | --- | --- | --- | --- | --- | --- | --- | --- | --- | --- |
| Genotype | 22 | 17.9 | *** | 9.6 | ns | 8.9 | * | 19.7 | *** | 11.1 | * |
| **Genotype sum of squares partition by *Ppd-A1*** | | | | | | | | | |  |  |
| *Between Ppd-A1* | *2* | *3.4* | *ns* | *2.0* | *ns* | *2.6* | *ns* | *4.4* | *ns* | *0.3* | *ns* |
| *Within Ppd-A1* | *20* | *14.5* | ***** | *7.6* | *ns* | *6.3* | *** | *15.3* | **** | *10.8* | *** |
| **Genotype sum of squares partition by *Ppd-B1*** | | | | | | | | | |  |  |
| *Between Ppd-B1* | *1* | *1.9* | *ns* | *0.0* | *ns* | *0.2* | *ns* | *0.2* | *ns* | *0.2* | *ns* |
| *Within Ppd-B1* | *21* | *16.0* | ***** | *9.6* | *ns* | *8.7* | *** | *19.5* | ***** | *10.9* | *ns* |
|  |  |  |  |  |  |  |  |  |  |  |  |
| Site x Genotype | 44 | 9.1 | *** | 12.6 | ns | 6.9 | ns | 11.0 | *** | 12.2 | ns |
| **Site x Genotype sum of squares partition by *Ppd-A1*** | | | | | | | | | |  |  |
| *Between Ppd-A1 x Site* | *4* | *0.7* | ns | *0.2* | ns | *1.6* | *ns* | *2.1* | *ns* | *2.8* | *ns* |
| *Within Ppd-A1 x Site* | *40* | *8.4* | *** | *12.4* | ns | *5.4* | *ns* | *8.9* | ***** | *9.4* | *ns* |
| **Site x Genotype sum of squares partition by *Ppd-B1*** | | | | | | | | | |  |  |
| *Between Ppd-B1 x Site* | *2* | *0.9* | ns | *0.3* | ns | *0.8* | *ns* | *0.3* | *ns* | *0.2* | *ns* |
| *Within Ppd-B1 x Site* | *42* | *8.2* | *** | *12.3* | ns | *6.1* | *ns* | *10.7* | ***** | *12.0* | *ns* |
| GDD: growing degree-days. ns: non-significant; **P*<0.05; ***P*<0.01;****P*<0.001. | | | | | | | | | |  |  |
