# Supplementary material for: Effect of Ppd-A1 and Ppd-B1 Allelic Variants on Grain Number and Thousand Kernel Weight of Durum Wheat and Their Impact on Final Grain Yield
Source: Front Plant Sci. 2018 Jun 29;9:888. doi: 10.3389/fpls.2018.00888 (PMC6033988; doi:10.3389/fpls.2018.00888)
Supplement: TABLE S5 — Mean values across 3 sites and 3 years for pre-flowering phenological phases for each Ppd-A1 and Ppd-B1 allele, expressed as growing degree-days (GDD). The number of genotypes carrying each allele is shown in Table 2. Different letters between alleles at each gene indicate differences according to LSD test at P < 0.05. [file Table_5.DOCX]

Supplementary Material

Effect of *Ppd-A1* and *Ppd-B1* Allelic Variants on Grain Number and Weight of Durum Wheat and their Impact on Final Grain Yield

Jose M. Arjona, Conxita Royo, Susanne Dreisigacker, Karim Ammar, Dolors Villegas^*^

***Correspondence:** Dolors Villegas: dolors.villegas@irta.cat

# Supplementary Table

| **Supplementary Table 5.** Mean values across 3 sites and 3 years for pre-flowering phenological phases for each *Ppd-A1* and *Ppd-B1* allele, expressed as growing degree-days (GDD). The number of genotypes carrying each allele is shown in Table 2. Different letters between alleles at each gene indicate differences according to LSD test at *P*<0.05. | | | | | | | | | | | |
| --- | --- | --- | --- | --- | --- | --- | --- | --- | --- | --- | --- |
| **Gene** | **Alleles** | **GDD emergence-double ridge (ºC)** | | **GDD double ridge - terminal spikelet (ºC)** | | **GDD terminal spikelet - booting (ºC)** | | **GDD booting - heading(ºC)** | | **GDD heading - flowering(ºC)** | |
| *Ppd-A1* |  |  | |  | |  | |  | |  |  |
|  | *Ppd-A1b* | 519 | a | 126 | a | 314 | a | 243 | a | 113 | a |
|  | *GS105* | 496 | a | 120 | a | 296 | a | 205 | a | 110 | a |
|  | *GS100* | 471 | a | 107 | a | 274 | a | 200 | a | 112 | a |
| *Ppd-B1* |  |  |  |  |  |  |  |  |  |  |  |
|  | *Ppd-B1b* | 519 | a | 122 | a | 305 | a | 226 | a | 110 | a |
|  | *Ppd-B1a* | 493 | a | 120 | a | 298 | a | 218 | a | 112 | a |
|  |  |  |  |  |  |  |  |  |  |  |  |
